# Supplementary material for: Comprehensive Annotation and Functional Exploration of MicroRNAs in Lettuce
Source: Front Plant Sci. 2021 Dec 24;12:781836. doi: 10.3389/fpls.2021.781836 (PMC8739914; doi:10.3389/fpls.2021.781836)
Supplement: Supplementary file 2 [file Data_Sheet_8.ZIP › GSM2230747.plot/Lsa-miRN1699a_Lsat_1_v5_gn_5_67060.1_48_TPlot.pdf]

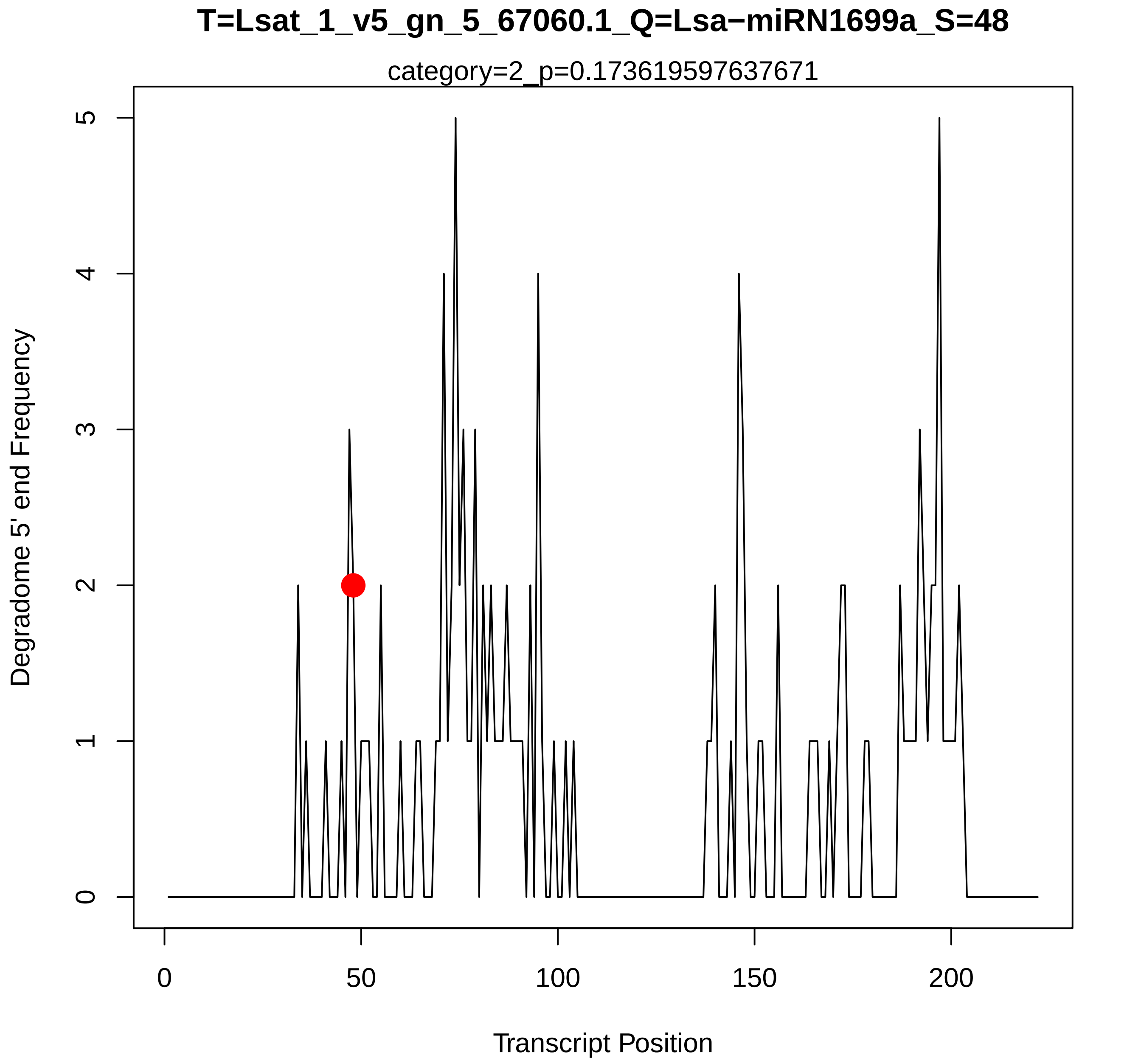
T=Lsat_1_v5_gn_5_67060.1_Q=Lsa−miRN1699a_S=48
category=2_p=0.173619597637671
Degradome 5' end Frequency
Transcript Position
0
1
2
3
4
5
0
50
100
150
200
